# What Would I Do if I Was an Emergency Physician? A Tool To Generate Discussion

**DOI:** 10.1007/s40670-025-02282-2

**Published:** 2025-01-23

**Authors:** Rob Eley, Diann Eley, Charley Greentree

**Affiliations:** 1 Emergency Department, Princess Alexandra Hospital (PAH), Brisbane, Queensland Australia; 2 PAH-Southside Clinical Unit, Faculty of Medicine, The University of Queensland, Brisbane, Australia; 3 Medical School, The University of Queensland, Brisbane, Australia; 4 Emergency Medicine, Redcliffe Hospital, Redcliffe, Queensland Australia; 5 Retrieval Services Queensland, Brisbane, Queensland Australia

**Keywords:** Medical education, Decision-making, Risk perception, Ambiguity tolerance, Reflective thought, Junior doctors, Professional development

## Abstract

Although safety is imperative in health care, medical practice carries a degree of uncertainty which in turn introduces risk, and perception of risk influences behaviour. Doctors worldwide are guided by professional practice frameworks, yet the perception of risk is subjective, meaning that individuals make their own judgements on its severity and consequences in ambiguous situations. The intent of this study was to develop a self-directed learning tool for medical students and doctors in training to prompt reflection on “what would I do” scenarios in ambiguous clinical emergency situations. We present the development and testing of scenarios based on the Australasian College for Emergency Medicine’s education and professionalism domains for use as a reflection tool in small group learning activities.

## Background

More than one interpretation of a clinical situation (i.e. ambiguity) is ubiquitous in medical education and practice. Tolerance for ambiguity remains a topic of concern in medical education [1, 2]. This is due to individual and subjective interpretation of ambiguous medical situations and its association with judgements around a degree of risk [3]. Every individual will make their own judgements on the severity of a risk, and this is influenced by the uncertainty of the situational context, the importance of the situation, and the individual’s emotions, mood, personality, and previous experience. Educators strive to improve students’ and trainee doctors’ ability to reflect on difficult/complex situations and decision-making where ambiguity is prominent.

Risk perception influences behaviour [4]; however, from the field of psychology, it is known that the value individuals put on risk also affects behaviour. This will occur even in an environment where the delivery of safe practice is prescribed practice. As an example, policy stipulates that gloves must be worn and doctors are aware of the risks of not wearing gloves — but still do not wear them all the time. The risks are the same, but the value that they put to the same risk differs among individuals. Recognition of the uncertainty of decision-making involving risk is relatively obvious with respect to safe practices (e.g. avoiding sharps injuries), however within clinical decisions or patient-clinician communication practices can be less obvious.

This study was undertaken to generate scenarios within the Australasian College for Emergency Medicine’s (ACEM) professional domains [5] (see Table 1). Each scenario provides various levels of ambiguity that require a judgement on the proposed action or behaviour. The aim was to develop a self-directed learning tool for medical students and doctors in training on how they perceive ambiguous situations in an emergency medicine clinical environment and how they would act in that situation. As a personal or group exercise for future trainees, the aim is to promote discussion and contemplative thought.

**Table 1:** Student and doctor responses

ACEM* domains for educational and training framework	Student** (%)			Doctor** (%)		
	Unlikely	Likely	Unsure	Unlikely	Likely	Unsure
**Scholarship and teaching**						
The ED consultant not disclosing to the patient that the intern is doing the procedure for the first time	47.2	44.9	7.9	42.0	54.0	4.0
**The ED consultant allowing the intern to attempt the intubation**	30.3***	**58.4**	11.2	80.0***	**20.0**	0.0
The ED consultant supervising the junior doctor managing a complex/difficult airway	14.6	78.7	6.7	30.0	64.0	6.0
The ED consultant allowing a registrar who is competent at ICC insertion to place an ICC in a morbidly obese patient without direct supervision	29.2	55.1	15.7	50.0	46.0	4.0
The junior doctor accompanying the consultant to a family conference to break bad news	6.7	84.3	9.0	4.0	94.0	2.0
**Communication**						
**Open disclosure of a child’s missed fracture to their parent over the phone**	**38.2**	**39.3**	**22.5**	**2.0**	**94.0**	**4.0**
**The ED doctor telling a family that their loved one will not survive the admission**	**39.3**	**44.9**	15.7	**10.0**	**88.0**	2.0
Talking to the family of a critically unwell patient, who have unrealistic expectations about the resuscitation of their loved one	5.6	88.8	5.6	2.0	96.0	2.0
Informing the mother who is interstate that their child is deceased from trauma	15.7	61.8	22.5	8.0	78.0	14.0
**Writing a police statement for a patient you saw 4 years earlier but you do not remember in detail**	**83.1**	**4.5**	12.4	**16.0**	**70.0**	14.0
**Health advocacy**						
The ED doctor notifying child services about a single mother of 2-year-old twins, who has presented to ED intoxicated with amphetamines	10.1	77.5	12.4	10.0	88.0	2.0
Calling the hospital executive/ED director when you believe the inpatient unit’s standard of care is suboptimal	41.6	43.8	14.6	32.0	62.0	6.0
Performing a tobacco smoking intervention on a young smoker in ED	23.6	67.4	9.0	42.0	52.0	6.0
**Calling an inpatient consultant to address their registrar’s attitude towards patients and staff in ED**	34.8	**51.7**	13.5	18.0	**78.0**	4.0
Stopping an actively bleeding wound before PPE can be applied	53.9	31.5	14.6	56.0	38.0	6.0
**Leadership and management**						
**The ED trainee who is transporting an unstable patient to the catheter laboratory for reperfusion therapy**	**49.4**	**24.7**	**25.8**	**20.0**	**74.0**	**6.0**
Removing an underperforming registrar from night shift	24.7	38.2	37.1	30.0	50.0	20.0
Directly admitting a patient to the ward against the wishes of the inpatient registrar	46.1	20.2	33.7	40.0	52.0	8.0
Telling your colleague that they made a misdiagnosis	13.5	68.5	18.0	14.0	82.0	4.0
**Removing an abusive patient from ED before completing their clinical assessment**	41.6	**28.1**	30.3	20.0	**66.0**	14.0
**Professionalism**						
**Prescribing antibiotics for a colleague after an informal undocumented consultation**	69.7	**20.2**	10.1	46.0	**44.0**	10.0
**Caring for the child of your boss in ED**	**38.2**	**43.8**	18.0	14.0	**80.0**	6.0
Directly being involved in caring for your family member in ED	80.9	12.4	6.7	94.0	4.0	2.0
Quitting your contract because you got offered a better job	24.7	47.2	28.1	44.0	44.0	12.0
Writing a medical certificate for a sick day for a friend of yours after the event, when you didn’t see them unwell	73.0	14.6	12.4	88.0	8.0	4.0
**Prioritisation and decision-making**						
**Sending the febrile 18-month-old with no rash home from ED without a definite diagnosis**	**65.2**	22.5	12.4	28.0	**68.0**	4.0
Discharging the 25-year-old female from ED with a positive pregnancy test, PV bleeding, normal abdominal and pelvic examination for follow up and ultrasound the next day	**52.8**	**28.1**	19.1	**30.0**	**58.0**	12.0
**Discharging a 19-year-old female who is not actively suicidal after presenting with an impulsive overdose**	38.2	**43.8**	18.0	26.0	**66.0**	8.0
Notifying authorities of a man who self-discharged from ED after presenting with a seizure whilst he was driving	7.9	85.4	6.7	24.0	74.0	2.0
**Discharging a 65-year-old man with moderate left iliac fossa pain and a past history of diverticulitis on oral antibiotics with no imaging**	**80.9**	**6.7**	12.4	**48.0**	**46.0**	6.0
ACEM* domains for education and professional framework						
**Medical expertise**						
**Performing a procedural sedation in ED on a non-fasted 4-year-old with a facial laceration**	**51.7**	**7.9**	**40.4**	**26.0**	**62.0**	**12.0**
**Repairing a laceration that crosses the vermillion border in ED on an adult’s face**	34.8	**13.5**	**51.7**	24.0	**76.0**	**0.0**
**Not performing a CT head on a patient on aspirin who suffered a head strike with no loss of consciousness**	70.8	**12.4**	16.9	52.0	**42.0**	6.0
**Exploring a penetrating thoracic wound in the Emergency Department**	**47.2**	16.9	36.0	**70.0**	14.0	16.0
Allowing an intoxicated patient with possible minor head injury (not witnessed) to self-discharge	77.5	11.2	11.2	62.0	20.0	18.0
**Teamwork and collaboration**						
**The ED consultant allowing the trainee to team lead a major resuscitation**	**47.2**	**38.2**	14.6	12.0	**82.0**	6.0
Complaining to the nurse in charge about what you feel is inappropriate triaging	32.6	55.1	12.4	48.0	48.0	4.0
**Speaking up if you feel that a senior colleague’s management of a patient is suboptimal**	**36.0**	**51.7**	12.4	**10.0**	**74.0**	16.0
**Telling a colleague to see more patients if they are working slowly**	**64.1**	**12.4**	23.5	**44.0**	**38.0**	18.0
Participating in team debrief after a clinical error in resuscitation	4.5	89.9	5.6	2.0	96.0	2.0

^*^ACEM, Australasian College for Emergency Medicine’s (ACEM) domains for education and professional framework

^**^Students were from years 1 to 4 of a 4-year MD program. Doctors consisted of interns (year 1 post-MD), house officers (year 2–3), registrars in training (years 4–8), and consultants (Fellows of the Australasian College of Emergency Medicine)

^**^Statements for which disparate views between doctors and students were > 20% are presented in **bold**

## Activity

This project was undertaken within the emergency department in a large metropolitan teaching hospital in Queensland, Australia. We recruited a convenience sample and 15 of the consultants working in the same emergency department as the author (CG) volunteered to assist in the development of clinical scenarios which offered ambiguity. In a stepwise process, using focus groups and nominal group technique, six of the consultants generated 12 emergency medicine-related statements for each of the eight domains of the ACEM Educational and Training Framework [5] that would stimulate a respondent to consider their behaviour. Thereafter, nine additional consultants each selected their top five statements from the 12 generated statements in each of the eight domains. The number of selections for each statement in each domain was counted and the resulting top five in each domain were used for the final tool of 40 statements.

The evaluation phase aimed to determine the level of disparity in response choice between medical students and doctors. Medical students (years 1–4, ~ 500) and emergency doctors (~ 250) across the health service were invited to complete the online survey via a link within the invitation email. All 40 statements were randomised with the RAND function in Excel. The online survey used a 7-point Likert scale (from extremely unlikely to extremely likely) in response to what action would be chosen for each statement.

Participant information within the survey preamble stated there were 40 statements of action and asked the participant to reflect on how unlikely/likely/unsure they agreed to the action described. Participants were encouraged to answer each statement thoughtfully from the viewpoint of their current role, knowledge, and experience. The study received ethics exemption from the Metro South Human Research Ethics Committee (HREC/15/QPAH/202). Completion of the survey was taken as consent.

## Results

**Fig. 1:**
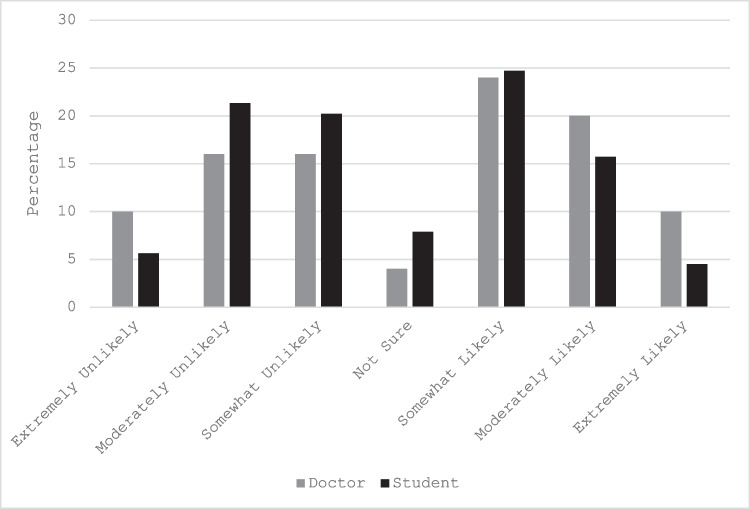
Example of similar responses between doctors and students to the statement *The ED consultant not disclosing to the patient that the junior doctor is doing the procedure for the first time*

**Fig. 2:**
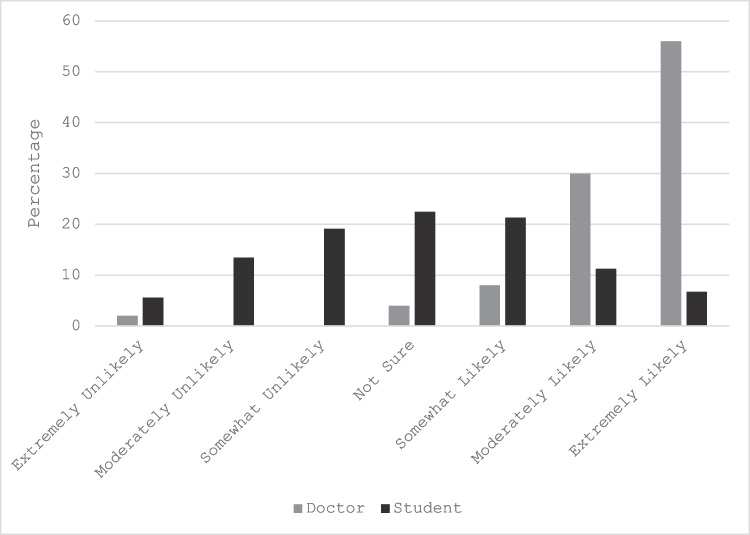
Example of widely different responses between doctors and students to the statement *Open disclosure of a child’s missed fracture to their parent over the phone*

All 139 responses (response 20% overall) from the 89 students and 50 doctors were complete and the response proportions for each statement within the unlikely/likely/unsure categories are presented in Table 1. Although some statements elicited similar responses from doctor and student cohorts, e.g. *The ED consultant not disclosing to the patient that the junior doctor is doing the procedure for the first time* (Fig. 1), disparate views were found among all domains and in almost half (19/40) of the 40 statements. For example, the statement regarding *Open disclosure of a child’s missed fracture to their parent over the phone* (Fig. 2) illustrates 94% of doctors indicated that they would follow this stated course of action while fewer than 40% of students would do so. While these differences between cohorts were observed, no clear pattern was identified within the year of student or level of doctor.

For several statements, the doctors themselves were not in agreement. The statement *The ED consultant allowing a registrar who is competent at ICC insertion to place an ICC in a morbidly obese patient without direct supervision* generated almost equal likely and unlikely responses. This level of disagreement was shown to be related to experience, with over two-thirds of the consultants stating this was unlikely, in contrast to three times as many more junior doctors saying it was likely.

Divergent views among doctors were also seen for *Prescribing antibiotics for a colleague after an informal undocumented consultation*. However, in this case, years of experience were not evident within the results. For example, from the 31 consultants, all with at least 10 years of training post-graduation, an equal number (15 vs 15) provided a likely or unlikely answer.

Differences between student responses across the years of medical school were only seen for 3 of the 40 statements.

- *Writing a police statement for a patient you saw 4 years earlier and do not remember in detail* — all 4 years of student considered this was unlikely with year 3 and 4 students being the most adamant that they would not do this. In complete contrast, doctors were adamant that they are likely to do this.
- *Calling the hospital executive/ED department director when you believe the inpatient unit’s standard of care is suboptimal —* none of the year 1 students stated they would be likely to call the executive. In contrast, 40% of years 2–4 would.
- *Directly being involved in caring for your family member in ED —* a quarter of year 1 and 2 students thought this was likely, as opposed to none of the year 3 and 4 students whose response was almost identical to that of the doctors.

## Discussion

The responses of the participants in this study illustrate, that as expected, the perception of risk is influenced by many factors and ultimately subjective [4, 6], with individuals making their own judgements on its severity and consequences of a risk. The variability that was seen supported the value of the tool as one for discussion and reflection.

It was never the intention to develop a tool for the purpose of assessment, but rather one for reflection and discussion. Self-awareness, insight, and reflective process are all mechanisms to enable value propositions to be evaluated. The aim of this project was to develop a tool to assist in this process. The resultant tool may be helpful for students, medical staff, and educators to understand why they tend towards certain behaviours and attitudes related to decision-making across the various domains of professional practice.

Although the statements were generated by emergency doctors within the setting of emergency departments, most of the statements have applicability in other clinical settings. In a similar vein to the scenarios presented in a multiple mini-interview, the statements recognise non-cognitive qualities such as maturity, teamwork, moral judgement, empathy, reliability, and communication skills. The clinically based statements provide a medical context. Application of this tool may be used by students and junior doctors as a reflective exercise to build self-awareness of their comfort with uncertainty and propensity for risk. For educators, it may help target support and training for staff to augment their confidence to the best advantage for their personal safety and that of their patients.

## Data Availability

The data that support the findings of this study are available from the corresponding author upon reasonable request.
